# United and Joyous

**Published:** 1918-07-06

**Authors:** 


### The Hospital

**The Workers' Newspaper of Administrative Medicine and Institutional Life, Administration, National Insurance and Health.**

No. 1674, Vol. LXIV. SATURDAY, JULY 6, 1918. PRICE TWOPENCE

### UNITED AND JOYOUS.

The year 1918 will go down to history as one of the greatest, or possibly the greatest, year in the calendar of civilised mankind. It is the year when the unity of the British and American peoples has been consummated and made eternal by the union of all their forces in the defence of the freedom of mankind. The blood of their bravest and best has been commingled in this defence of principles which must be upheld and maintained, or the world would cease to be a place where free men and women could continue to live in any right meaning of the word. The basis of unity is the awakening of the peoples to the recognition of the mission which has been imposed upon them and their Allies. Circumstances have made it clear to the free and independent peoples of the world that the call to stand together, do their utmost, and give of their best, is a call recognised as coming from the Highest Powers to Whom we all look for salvation and immortality. Nothing more wonderful has ever happened than this conviction which has come to the hearts of the sailors, the soldiers, the airmen, and all warriors fighting with the Allies in the cause of freedom. The youth and manhood of every nation engaged in the upholding of righteousness, justice, and mercy, in the defence and protection of the women, the children, the aged, the defenceless, and in the convinced maintenance of the highest principles which we can conceive, shine forth as a beacon light which will permeate the ages to come and make the present war and the unity of the English-speaking peoples glorious so long as the world lasts.

For forty years it has been our privilege to be in constant and increasing touch with the life and people of the United States, who have been among the most active workers and fruitful producers of results which have proved momentous for good in the developments, uplifting, increasing life and growth, which have tended to bring our cousins across the water to a position of leadership and creation. These have proved far-reaching in importance to their people and stimulating and helpful to those of us in the Old Country who have regarded them as united to us by blood, by sentiment, by everything which is best in life. In the particular fields covered by this journal, the interrelation, exchange of ideas, cordial co-operation and continuous counsel, inquiry, and fellowship, have contributed, possibly, more than anything else, to the momentous developments in science, in treatment, in discovery, in all things which have an intimate bearing upon the improvement of the conditions, under which our people live, work and have their being. Thus have come to us, as Mr. Balfour has well said, the perception of the true development of free institutions, the consciousness of common kinship and common ideals, with all the considerations which ought to bind us together, which have bound us together, and which day by day, year by year, generation by generation, century by century, are going to bind us still closer together in the future.

Hence it is that we found ourselves joining with our American friends in celebrating the 4th July, 1918. Mr. Balfour is right in declaring we are working together in all the freedom of great hopes and great ideals. And these hopes and these ideals we have not learnt from each other. We have them in common from a common history and a common ancestry. We have not learnt freedom from our American cousins, nor they from us. We both spring from the same root. We both cultivate the same great ends. We both have the same hopes as regards the future of Western civilisation; and now that we have found ourselves united in this great struggle against a Power, which, if it be allowed to prevail, is going to destroy the very roots of that Western civilisation, from which we all draw our strength, are not we bound together for ever? We believe, too, that our descendants, looking back upon this unique episode in the history of the world, will agree that, its most beneficent and permanent result was that which brought together and united for a common purpose, in one common understanding, the two great branches of the English-speaking race. So it is a fact that wherever our cousins and ourselves mingle since we joined hands in this war, we form a joyous, confident, uplifted, buoyant company, recognising our mission and resolved to accomplish it or perish.

